# Improving Awareness of Oman Medical Specialty Board Training Bylaws Among First-Year Pediatric Residents Using a Flipped Classroom Intervention: A Quality Improvement Project

**DOI:** 10.7759/cureus.116397

**Published:** 2026-09-16

**Authors:** Saniya Al Husaini, Abdul Hamid Al Hinai, Majan Al Siyabi, Sawsan Alnabhani, Mazoon AlSaadi

**Affiliations:** 1 Child Health, Royal Hospital, Muscat, OMN; 2 Genetic and Developmental Medicine Clinic, Department of Genetics, Sultan Qaboos University Hospital, University Medical City, Muscat, OMN; 3 Pediatrics, Al Suwaiq Hospital, South Al Batinah, Al Suwaiq, OMN

**Keywords:** competency-based medical education, educational intervention, flipped-classroom, hospitalist resident education quality improvement, pediatric residency education, program evaluation, quality improvement projects, residency training, medical education

## Abstract

Background: Awareness of institutional training regulations is essential for safe and compliant residency practice. Among first-year residents in the Oman Medical Specialty Board (OMSB) pediatric residency program, limited familiarity with OMSB bylaws was demonstrated.

Local problem: Despite the availability of OMSB bylaws through institutional platforms, first-year pediatric residents demonstrated limited awareness and understanding of key training regulations, which may affect their ability to navigate residency requirements and access available support systems.

Methods: A quality improvement (QI) project was conducted among first-year pediatric residents. A flipped classroom intervention was implemented using pre-session learning materials followed by an interactive group discussion. Awareness of OMSB bylaws was assessed using a pre- and post-intervention questionnaire. Outcome measures included change in knowledge scores.

Results: Among the 31 eligible residents, all participants completed both the pre- and post-intervention assessments, yielding a 100% response rate. The mean knowledge score increased from 58.2% at baseline to 80.7% following the intervention, corresponding to an absolute improvement of 22.5 percentage points and a relative increase of 38.7%. Awareness scores were higher after the intervention in eight of the nine policy domains.

Conclusions: Knowledge scores improved following implementation of a flipped classroom intervention addressing OMSB bylaws among first-year pediatric residents. The intervention was delivered within existing teaching time with complete participation, indicating that the approach is feasible for residency onboarding programs.

## Introduction

Awareness of institutional policies is a critical component of residency training, as it provides a framework for professional protection, promotes patient safety, and supports resident advocacy and well-being. Residents occupy a dual role as trainees and as practising professional staff with limited administrative authority, requiring them to navigate complex legal, ethical, and administrative expectations. Without adequate understanding of governing policies, residents may be vulnerable to professional risks, reduced autonomy, and suboptimal engagement with institutional processes [1].

Training institutions typically attempt to standardize policy awareness through structured orientation programs, digital access to policy documents, and incorporation into educational activities. Despite these efforts, studies in medical education suggest that trainees frequently demonstrate limited awareness and retention of institutional policies [2]. Several factors may contribute to poor awareness of institutional policies among residents. Policy documents are often lengthy, numerous, and infrequently revisited after orientation. Communication is commonly limited to emails or document dissemination with minimal interactive discussion or reinforcement. In addition, competing clinical responsibilities and limited protected time may reduce resident engagement with administrative and policy-related content [3].

The flipped classroom model in medical education represents a shift from traditional teacher-led lectures to student-centered active learning. In this pedagogical framework, learners acquire foundational knowledge through pre-class materials like videos or readings, allowing in-class time to be dedicated to interactive problem-solving and collaborative exercises [4]. Research results indicate that this method significantly improves academic performance, critical thinking, and student engagement compared to conventional instruction [5]. Despite its advantages, the flipped classroom model presents substantial challenges for both educators and students. This includes a substantial requirement in initial investment of time and resources to create high-quality pre-class materials (like videos) and design interactive in-class activities. Moreover, it depends on student accountability and readiness, which is not always guaranteed [6].

This quality improvement (QI) project aimed to improve awareness of nine key institutional policies among first-year pediatric residents using a flipped classroom educational intervention. The improvement aim was defined at the outset as a SMART (Specific, Measurable, Achievable, Relevant, and Time-bound) objective, in accordance with standard QI practice, and was set as an absolute increase of at least 20 percentage points in the mean awareness score between the pre- and post-intervention assessments, to be achieved within four weeks of the intervention. This figure was adopted by the project team as a meaningful and attainable improvement target for the program rather than as a statistically derived threshold.

## Materials and methods

Setting and context

This QI initiative was conducted within the pediatric residency program at Oman Medical Specialty Board (OMSB), the national authority responsible for postgraduate medical education and specialty certification in Oman. OMSB is accredited by the Accreditation Council for Graduate Medical Education International (ACGME-I) [7] and oversees more than 30 residency and fellowship training programs across multiple specialties.

In September 2024, 31 first-year pediatric residents were enrolled in the residency program and constituted the study population. Institutional policies and bylaws are accessible through the OMSB New Innovation Platform as open-source resources [8]; however, review of these materials is encouraged but not mandatory.

Intervention(s)

This QI project was designed using the Plan-Do-Check-Act (PDCA) cycle framework to assess and improve first-year pediatric residents' awareness of nine key OMSB policies. The selected policies included: Residency Performance and Well-Being, Needle Stick or Penetrating Injury, Duty Hours, Withdrawal Policy, Remediation Criteria, Sick Leave Policy, Scientific Leave, Annual Leave, and Compensation for Public Holidays Policy. These nine policies were selected on the recommendation of the OMSB pediatric training department, based on its experience of the administrative difficulties most frequently encountered by first-year residents and on the observation that, although the bylaws are openly accessible, residents were not routinely reviewing them. Table 1 summarizes the key aspects of the nine policies. These policies were sent to residents via email as pre-class study material.

**Table 1:** Summary of the key aspects of the nine OMSB policies Adapted by the authors from the OMSB Training Bylaws [8]. Content is the authors' original paraphrase of publicly available institutional policy documents; no material was reproduced from a copyrighted publication. OMSB: Oman Medical Specialty Board

Policy	Key aspect
Withdrawal Policy	Residents may withdraw within the first 4 months with replacement allowed and exit after completion. After 4 months, withdrawal is delayed until year-end, with a one-year ban from OMSB programs.
Resident Performance and Well-Being	The OMSB Performance and Wellness Section supports residents' academic performance and overall well-being by providing confidential assessment, psychological and medical support, external referrals, and career guidance. It also advises training committees, promotes educational interventions, and ensures early identification and management of residents experiencing training difficulties.
Needle Stick or Penetrating Injury	Residents must immediately report needle-stick or body fluid exposure to Infection Control and follow hospital protocols. Infected residents are referred to specialists for evaluation, counseling, and further OMSB guidance.
Duty Hours	Resident duty hours are capped at 80 hours/week with one free day in seven. In-house calls are limited to one every 4 days, shifts ≤24 hours, and weekend calls twice per block. At-home call counts toward rest hours, must ensure rest requirements, and is not permitted for first-year pediatric residents.
Remediation Criteria	Remediation is required for residents with two unsatisfactory evaluations in a year, failure of key exams after second attempt, or remediable concerns in professional conduct.
Sick Leave Policy	Residents must notify supervisors and program administration when on sick leave, submit leave and return forms with a medical certificate within two weeks, and compensate any sick leave exceeding seven days before training completion certification.
Scientific Leave Policy	Residents may receive up to 10 days scientific leave annually for conferences or exams with proof of attendance. Requests must be submitted 3 months in advance, require supervisor and program director approval, and include return documentation within two weeks.
Compensation for Public Holiday Policy	Residents may receive up to 10 days compensation leave for public holidays worked. Regular on-call duties are excluded. Approval requires documentation within 2 weeks, and leave is scheduled by the training program.
Annual Leave Policy	Residents are entitled to 30 annual leave days plus compensatory holidays, with no carryover. Leave requires advance planning, multi-level approval, and proper documentation, ensuring training continuity and minimum rotation attendance.

The educational intervention utilized a flipped classroom approach, whereby residents reviewed the relevant educational materials before participating in an in-person learning session. Policy documents were distributed electronically via email one week prior to the session to facilitate pre-session preparation. The pre-session materials consisted of the relevant sections of the OMSB Training Bylaws covering the nine selected policy domains; no pre-recorded lectures, videos, or other multimedia modules were used. The face-to-face session was conducted during the first-year pediatric residents' off-service teaching day and lasted approximately one hour. Residents were divided into small groups, with each group assigned a hard copy of specific policy documents and tasked with presenting and explaining the content to their peers. A faculty facilitator guided the session, promoting interactive discussion, encouraging participant engagement, and providing clarification of policy content as needed.

Study of the intervention

The initiative was conducted over an eight-week period from December 2024 to January 2025, encompassing baseline assessment, the flipped classroom intervention, and post-intervention assessment. A pre-post study design was used to evaluate the intervention. All 31 first-year pediatric residents enrolled in the program at the time were included, and all completed both assessments. The questionnaire was developed by the study team directly from the text of the OMSB Training Bylaws. Each item was written as a multiple-choice question with a single pre-defined correct answer traceable to a specific bylaw provision. The draft instrument was reviewed by pediatric education faculty and OMSB training representatives prior to implementation; following this review the number of items was reduced and the questionnaire was restructured around the nine policy domains, yielding the final 31-item instrument. Content validity was established through this review process. Formal psychometric evaluation was not undertaken: internal consistency, test-retest reliability, item difficulty, and item discrimination were not assessed, and no alternate or parallel form of the questionnaire was developed. The full instrument is provided in Appendix 1. This project was reported in accordance with the Standards for Quality Improvement Reporting Excellence (SQUIRE 2.0) guidelines [9].

Baseline knowledge was assessed using a 31-item multiple-choice questionnaire administered via Google Forms prior to any project-related distribution of policy materials, that is, before both the pre-session email and the in-person session. At the time of baseline assessment, the OMSB training bylaws were, as for all residents, freely accessible through institutional platforms but not designated as required reading; the baseline questionnaire was therefore intended to capture residents' existing awareness under routine conditions of open access. The flipped classroom intervention was planned in response to these baseline findings. Policy documents were subsequently distributed by email one week before the in-person session as standard pre-class preparation, and the same questionnaire was redistributed four weeks after the session to measure changes in residents' awareness and understanding of the policies. The same instrument was administered at both time points so that pre- and post-intervention scores referred to identical bylaw content and were directly comparable; the implications of this for testing effects are addressed in the study limitations.

Measures and analysis

The primary measure was the change in residents' knowledge scores between pre- and post-intervention assessments. Performance was quantified based on correct responses to the 31-item questionnaire, reflecting awareness of the nine OMSB policies.

Each of the 31 questionnaire items was scored dichotomously, with one point awarded for a correct response and zero points for an incorrect or unanswered response. Item scores were summed to yield a total raw score (range 0-31), which was converted to a percentage (number correct/31 × 100) for both the overall score and for each of the nine policy domains, to allow comparison across domains containing unequal numbers of items. Higher percentage scores indicate greater awareness and understanding of OMSB training bylaws.

Data were analyzed using Excel. Descriptive statistics were used to summarize participant characteristics and questionnaire scores. Pre- and post-intervention mean knowledge scores across the nine policy domains were compared using a paired-samples t-test, with the policy domain as the unit of analysis. As set out below, this comparison is reported as a descriptive and exploratory analysis rather than as a test of resident-level change. No formal power calculation was performed: the project included the entire eligible cohort rather than a sample determined by a target sample size, and the 20 percentage-point figure was set as a QI aim rather than as a hypothesis to be tested at a specified level of statistical power.

The nine policy domains were represented by unequal numbers of questionnaire items, ranging from a single item (Needle Stick or Penetrating Injury; Resident Performance and Well-Being) to six items (Duty Hours; Sick Leave Policy). The domain was therefore used as the unit of analysis so that each policy area contributed equally to the overall knowledge score, irrespective of how many individual items it comprised, reflecting the operational aim of establishing whether awareness improved across each of the nine bylaw areas, each of which carries comparable practical consequence for a resident. The questionnaire had been organized around these domains on the advice of the faculty reviewers at the instrument development stage. The decision to conduct the statistical analysis at the domain level was, however, made after data collection rather than pre-specified in a formal analysis plan. This approach does not represent the paired resident-level structure of the data, and the resulting statistics describe variation across policy domains rather than across residents. Domain-level results are therefore reported as descriptive and exploratory, and item-level findings were used only to identify areas of persistent knowledge deficiency.

Ethical considerations

This project was conducted as a QI initiative within the OMSB Pediatric Residency Program. According to institutional policy, formal ethical approval was not required because the project evaluated an educational intervention aimed at improving program performance. Participation was voluntary, questionnaire responses were anonymized prior to analysis, and no patient data were collected.

## Results

A total of 31 first-year pediatric residents completed both the pre-intervention and post-intervention assessments, resulting in a 100% response rate. The 31-item questionnaire assessed awareness of nine key OMSB policy domains. Figure 1 presents the percentage of correct responses for each domain before and after the flipped classroom intervention.

**Figure 1:**
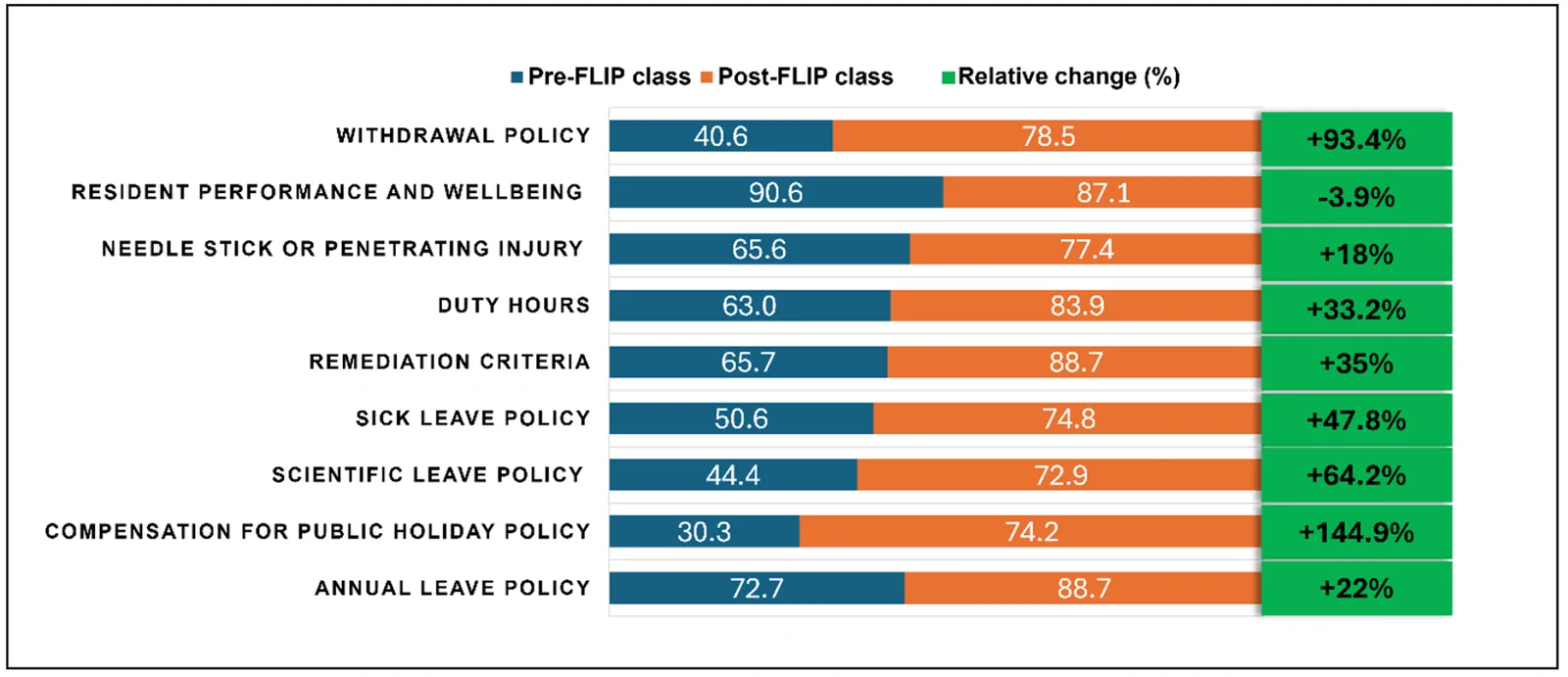
Comparison of pre-intervention and post-intervention knowledge scores (percentage of items answered correctly) across nine OMSB policy domains among first-year pediatric residents (N = 31) following the flipped classroom intervention. Relative percentage change for each domain is shown on the right. OMSB: Oman Medical Specialty Board

The overall mean knowledge score increased from 58.2% before the intervention to 80.7% after the intervention, representing an absolute improvement of 22.5 percentage points and a relative improvement of 38.7%, exceeding the project's aim of a 20 percentage-point improvement. The exploratory domain-level comparison is summarized in Table 2. As noted in the section Measures and Analysis above, these statistics describe variation across the nine policy domains rather than across individual residents and do not constitute a test of resident-level change.

**Table 2:** Exploratory domain-level comparison of pre-intervention and post-intervention knowledge scores across the nine OMSB policy domains (n = 9 domains). These statistics describe variation across policy domains, not across individual residents, and do not constitute a resident-level test. OMSB: Oman Medical Specialty Board; CI: Confidence interval

Measure	Value
Mean knowledge score, pre-intervention, %	58.2 ± 18.5
Mean knowledge score, post-intervention, %	80.7 ± 6.5
Mean difference (95% CI)	22.5 (11.8 to 33.3)
t(8)	4.83
p-value	0.0013

The largest improvements were observed in awareness of the Compensation for Public Holiday Policy (+43.9 percentage points, from 30.3% to 74.2%; relative improvement +144.9%), Withdrawal Policy (+37.9 percentage points, from 40.6% to 78.5%; +93.4%), and Scientific Leave Policy (+28.5 percentage points, from 44.4% to 72.9%; +64.2%).

Substantial improvements were also observed in Sick Leave Policy (+24.2 percentage points, from 50.6% to 74.8%), Remediation Criteria (+23.0 percentage points, from 65.7% to 88.7%), and Duty Hours (+20.9 percentage points, from 63.0% to 83.9%).

More modest improvements were noted in Needle Stick or Penetrating Injury (+11.8 percentage points, from 65.6% to 77.4%) and Annual Leave Policy (+16.0 percentage points, from 72.7% to 88.7%).

Awareness of Resident Performance and Well-Being decreased slightly from 90.6% to 87.1% (−3.5 percentage points; relative change −3.9%). Despite this small decline, baseline performance in this domain remained high both before and after the intervention.

Participant engagement was high, with all residents attending the in-person session and completing both assessments.

## Discussion

This QI initiative found that first-year pediatric residents' awareness of key OMSB bylaws was higher following a structured flipped classroom intervention. The findings are consistent with the view that active educational strategies may support resident understanding of institutional regulations more readily than passive dissemination of policy documents alone. To our knowledge, few studies have evaluated educational interventions specifically targeting awareness of residency training bylaws and administrative policies.

The flipped classroom model has been associated with significant improvements in both learner performance and engagement across medical and health professions education. Evidence from surgical residency programs demonstrates that adopting this model can enhance standardized examination outcomes, with mean percentile scores increasing substantially and fewer trainees performing at lower percentiles [10]. Similar benefits have been observed in pediatric residency training, where interns exposed to flipped classroom teaching showed statistically significant gains in knowledge compared to those receiving traditional instruction [11].

In pharmacy education, flipped classroom implementation has also led to notable academic gains, including higher final examination scores and an increased proportion of top-performing students [12]. Beyond immediate academic performance, the model supports improved knowledge retention, likely due to its emphasis on active learning strategies rather than passive information delivery [4].

In terms of engagement, learners consistently report a preference for flipped classroom approaches over conventional lectures. Trainees describe feeling better prepared for assessments and more involved in the learning process [13].

The flipped classroom promotes active participation by dedicating in-person sessions to discussion, application, and problem-solving, while foundational content is learned independently. This shift enhances peer interaction and redefines the educator's role as a facilitator rather than a lecturer. Importantly, the model also contributes to the development of essential professional skills, including communication, teamwork, and problem-solving [4].

The largest improvements were observed in policy domains with the lowest baseline awareness, suggesting that residents may benefit most from structured educational interventions in areas that are less frequently encountered during routine training. The findings highlight the importance of incorporating policy education into formal onboarding processes rather than relying solely on self-directed review of institutional documents.

Improved awareness of training regulations may reduce resident uncertainty, improve utilization of available support systems, and facilitate compliance with residency requirements. As awareness was reassessed four weeks after the session, these observations describe the short-term effect of the intervention; longer-term follow-up is required to establish retention and sustained educational impact.

This QI initiative has several limitations. The comparison was conducted at the level of the nine policy domains rather than individual residents, so the reported statistics describe variation across policy domains rather than across residents. The domain-level comparison should therefore be interpreted as descriptive and exploratory rather than as a test of resident-level change, and this analytical approach was adopted after data collection rather than pre-specified in a formal analysis plan.

The questionnaire was not validated beyond content review. Internal consistency, test-retest reliability, item difficulty, and item discrimination were not assessed, and as the primary outcome rests entirely on this instrument, its measurement properties remain uncharacterized. No alternate or parallel form was developed, and the same 31 items were administered at both time points, so a testing or recall effect may have contributed to the observed improvement. Formal pilot testing was not undertaken, as this is not a routine requirement for a QI project of this type.

The study was conducted in a single pediatric residency program and involved only one resident cohort, limiting the generalizability of the findings. As a QI project designed around the PDCA framework rather than an experimental or comparative study, no concurrent or historical control group was included, and no group received passive dissemination alone. The project, therefore, cannot establish that the flipped classroom approach was superior to passive distribution of policy documents, or that it was solely responsible for the observed change. Maturation and routine clinical exposure over the project period, and a Hawthorne effect whereby awareness of being assessed may have prompted independent review of the policies, cannot be excluded as contributors. The two-phase assessment design also does not allow the contribution of the emailed pre-session materials to be distinguished from that of the in-person session, and the observed improvement may reflect their cumulative effect. A three-phase assessment conducted at baseline, following distribution of the pre-session materials, and following the in-person session would allow these components to be evaluated separately and is recommended for future iterations, although advance distribution of materials is intrinsic to the flipped classroom method rather than a separable intervention.

Completion of pre-session preparation could not be verified. The project assessed knowledge outcomes only and did not evaluate behavioral change, policy adherence, or retention beyond the four-week follow-up interval; durable retention, therefore, cannot be inferred.

## Conclusions

Knowledge scores improved following implementation of a flipped classroom intervention addressing key residency bylaws among first-year pediatric residents. The intervention was delivered within existing teaching time with complete participation, indicating that the approach is feasible in this setting. Integrating structured policy education into residency orientation programs may support residents' understanding of training requirements and engagement with available support systems, although the absence of a comparison group and the four-week follow-up interval limit the inferences that can be drawn. Future studies should evaluate longer-term knowledge retention, behavioral outcomes, and the applicability of this approach across other residency programs and specialties.

## Appendices

Appendix 1: OMSB bylaws awareness questionnaire

1. How many days of annual leave are residents entitled to each year?

A. 20 days B. 25 days C. 30 days D. 35 days

2. When should residents ideally submit their proposed annual leave?

A. Before the creation of the master rotation schedule B. At least 2 months before the leave date C. Anytime during the year

3. If a resident applies for leave after the master rotation schedule is finalized, how far in advance must they submit their application?

A. 1 month B. 2 months C. 3 months

4. What must a resident complete upon returning from annual leave?

A. Return from Leave Form B. Rotation Evaluation Form C. Annual Assessment Form D. Scientific Leave Form

5. What is the maximum number of days a resident can take as compensation for working during public holidays?

A. 5 days B. 7 days C. 10 days D. 15 days

6. What document must a resident submit to request compensation leave?

A. Leave form submitted to the Chief Resident B. Email to the OMSB Executive President C. Letter signed by the Program Director or Associate Program Director and Rotation Supervisor

7. Within how many weeks from the public holiday must the compensation leave request be submitted?

A. 1 week B. 2 weeks C. 4 weeks

8. What is the maximum number of days allowed for scientific leave during each academic year?

A. 5 days B. 7 days C. 10 days D. 15 days

9. How far in advance must a resident submit the scientific leave form?

A. 1 month before B. 2 months before C. 3 months before

10. What proof must a resident provide upon returning from scientific leave?

A. Personal statement B. Copy of conference agenda C. Certificate of Attendance or Examination Certificate

11. What form must a resident complete upon returning from scientific leave?

A. Leave Request Form B. Program Completion Form C. Examination Results Form D. Return from Leave Form

12. Within how many weeks must a resident submit the required documents after returning from scientific leave?

A. 1 week B. 2 weeks C. 4 weeks

13. Who must a resident notify when taking sick leave?

A. Rotation Supervisor only B. Rotation Supervisor and Program Administrator C. Rotation Supervisor and Program Director/Associate Program Director D. All of the above

14. When should a resident notify the relevant personnel about their sick leave?

A. After returning from leave B. Only if the leave exceeds 7 days C. Before the leave starts and after returning from leave

15. What documents must a resident complete upon returning from sick leave?

A. Leave Form only B. Medical Certificate only C. Return from Leave Form and Medical Certificate D. All of the above

16. From where must the Medical Certificate be issued?

A. Any clinic B. Authorized hospital

17. Within how many weeks must the required documents be submitted after returning from sick leave?

A. 1 week B. 2 weeks C. 4 weeks

18. What must be compensated if a resident’s sick leave exceeds 7 days?

A. Half of the rotation period B. One additional month C. The exact number of days missed D. The entire rotation

19. How many “Unsatisfactory/Below Expectations” evaluations in one academic year trigger remediation?

A. One B. Two C. Three D. Four

20. What happens if a resident fails the End-of-Year Examination twice?

A. No consequence B. Immediate termination C. Remediation is initiated

21. What is the maximum number of duty hours a resident can work per week, averaged over four weeks?

A. 60 hours B. 70 hours C. 80 hours D. 100 hours

22. How many days per week must a resident have free from all patient care and educational obligations, averaged over four weeks?

A. 0 days B. 1 day C. 2 days D. 3 days

23. What is the maximum number of in-house calls a resident can take per block?

A. 4 calls B. 5 calls C. 6 calls

24. How frequently can a resident take in-house calls?

A. Every 3 days B. Every 4 days C. Every 5 days

25. What is the maximum number of weekend calls allowed per block?

A. 1 call B. 2 calls

26. After completing 24 hours of continuous duty, how many additional hours can a resident remain on duty?

A. 2 hours B. 4 hours C. 6 hours D. 8 hours

27. Where should a resident report a needle-stick injury or body fluid exposure?

A. Emergency Room B. Infection Control Department C. Human Resources Department

28. Which section has been established to provide support for residents?

A. Residency Affairs Section B. Education Advisory Section C. Performance and Wellness Section

29. If a resident withdraws within the first 4 months of residency, what happens to their position?

A. The position remains vacant B. The position is permanently closed C. The resident must complete the academic year D. The program may replace the vacancy with a candidate from the waiting list

30. If a resident withdraws after the first 4 months of the academic year, when can they leave?

A. Immediately B. After 6 months of notice C. At the end of the academic year

31. How long is a resident prohibited from applying to any OMSB Residency Training Program after withdrawal?

A. 6 months B. 1 year C. 2 years D. No restriction
